# Development of a Synergistic Nanomaterial Scaffold Combining Silver Nanoparticles, Collagen, and Doxycycline for Enhanced Scar-Free Skin Regeneration

**DOI:** 10.7759/cureus.64875

**Published:** 2024-07-18

**Authors:** Chokkalingam Deepa, Selvaraj Bharathi, Poongazhalselvan Devagi, Baskaran Sivasankari, Umapathi Prakash, Kuppusamy Kavitha, Gopal Suresh, Arumugam Rajalakshmi, Balasubramanian Ramesh, Gajapathi Balaraman

**Affiliations:** 1 Research Department of Microbiology, Sri Sankara Arts and Science College, Kanchipuram, IND; 2 Department of Research Analytics, Saveetha Dental College and Hospitals, Chennai, IND; 3 Research Department of Biotechnology, Sri Sankara Arts and Science College, Kanchipuram, IND; 4 Department of Prosthodontics, Sri Venkateshwara Dental College and Hospital, Chennai, IND

**Keywords:** wound healing, doxycycline, collagen, delphinium denudatum, silver nanoparticles

## Abstract

Introduction

The efficacy of wound-healing treatments can be significantly enhanced through innovative combination therapies. This research investigates the wound-healing properties of a combination therapy involving silver nanoparticles (AgNPs) synthesized using Delphinium denudatum (Dd), bovine tendon collagen (BTC), and the antibiotic doxycycline (DOX) in Wistar albino rats. Each component has known therapeutic benefits: AgNPs possess antimicrobial properties, BTC aids in tissue regeneration, and DOX is an effective antibiotic. The synergy between these components is hypothesized to enhance wound closure, reduce inflammation, and promote scar-free healing.

Methods

The synthesis of DdAgNPs was carried out using Dd. The presence of AgNPs was confirmed by ultraviolet-visible (UV-Vis) spectroscopy and high-resolution transmission electron microscopy (HRTEM). The study was conducted on Wistar albino rats following ethical guidelines for animal research. The rats were divided into different groups to receive various treatments: DdAgNPs alone, BTC alone, DOX alone, combinations of two components, and the triple combination of DdAgNPs: BTC: DOX. Wound closure rates, epithelialization, and collagen deposition were monitored and recorded over time. Tissue samples from the wound sites were collected for histological analysis. Hematoxylin and eosin (H&E) staining was used to evaluate epithelialization and overall tissue architecture. Collagen deposition was assessed using Masson's trichrome staining. Additionally, the expression of cyclooxygenase-2 (COX-2) was measured as an indicator of inflammation.

Results

UV-Vis spectroscopy provided the characteristic surface plasmon resonance peak indicative of AgNPs, while HRTEM revealed the morphology and size of the nanoparticles, showing spherical particles with an average size of 35±10.42 nm. The combination therapy of DdAgNPs: BTC: DOX significantly enhanced wound closure compared to individual and dual-component treatments. This was evidenced by faster epithelialization and increased collagen deposition. The histological analysis showed that the triple combination treatment resulted in more organized tissue architecture and denser collagen fibers. Furthermore, the treatment led to a marked decrease in COX-2 expression, indicating reduced inflammation and potential for lower scar formation.

Conclusion

The synergistic application of DdAgNPs, BTC, and DOX presents a promising strategy for advanced wound healing and tissue regeneration. The combination therapy not only accelerates wound closure but also enhances the quality of healing by promoting epithelialization and collagen deposition while reducing inflammation. These findings offer a potential pathway for developing effective, scar-free healing solutions, highlighting the benefits of integrating multiple therapeutic agents in wound care.

## Introduction

The skin serves to safeguard the body from environmental hazards, and wounds disrupt normal tissue structure and function [1]. The body initiates healing through an inflammatory response, increasing collagen production and regenerating epithelial tissue [2]. Wound healing occurs through several stages, including hemostasis, inflammation, proliferation, and remodeling. Chronic wounds do not heal orderly and are often in a long-term inflammatory state. The goal of wound care is to expedite closure while minimizing pain, discomfort, and scar formation. A formulation combining antibacterial and cell growth-promoting action is needed, as modern dressings like hydrogels [3], hydrocolloids, and collagen films expedite wound healing [4].

Traditional silver nanoparticle (AgNP) preparation methods are expensive and energy-intensive and pose risks to people. Recent research focuses on green synthesis of nanoparticles for biological and medicinal applications. AgNPs due to their affordability, energy efficiency, and non-toxicity exhibit unique properties when combined with collagen, thereby creating a synergy that enhances their unique properties of wound healing [5]. AgNPs are synthesized from Delphinium denudatum (Dd) aqueous root extract, known as Jadwar, an important medicine in indigenous Indian medicinal systems, especially Unani [6]. The bitter root of Dd is used to treat poisoning, rheumatism, snakebite, opium, and other disorders [7-8].

Collagen acts as a key player in the wound healing process, because of its chemical characteristics, its ability to direct cellular activities, and its function in nucleation. It attaches to fibrotic cells, guides fibroblasts, and supports capillary growth, promoting the repair of damaged tissue [9-10]. The combination of bovine tendon collagen (BTC) and antibiotics accelerates wound healing, reduces complications, and suppresses bacterial colonization. Doxycycline (DOX), a chemically modified tetracycline derivative, cures infections, reduces inflammation, and increases the cytokines of the injury site [11]. Wistar rats are commonly used to evaluate skin wound healing and treatment success due to their availability, low cost, and small stature [12] and several wound healing investigations have used scratch models on the rat dorsal fin for wound healing studies [13]. In our previous work, the synthesis of AgNPs using an aqueous root extract of Dd and its antibacterial properties was demonstrated [8]. Hence the current investigation focused on exploiting the wound-healing capacity of a combination of DdAgNPs, BTC, and DOX in Wistar albino rats.

## Materials and methods

Extraction of collagen from bovine Achilles tendons

The bovine Achilles tendon was sourced from a slaughterhouse in Kanchipuram and was cleaned of sand and other debris under running water. The tendons were divided into tiny pieces and left to soak in 33% acetic acid for 72 h. Upon chopping them, 3% acetic acid was used to treat them. After diluting with double the volume of sterile water, a 5% NaCl solution (2x volume) was added. The mixture was then stirred with a sterile glass rod to precipitate the collagen. Collagen was isolated through sequential acidic and saline washes. Initially, the material was adhered to a glass rod and treated with a concentrated acetic acid solution (33%). Subsequently, a lower concentration salt solution (5% NaCl) was introduced while stirring with the rod. This detachment-reattachment process aimed to purify the collagen. Following multiple washes with sterile water, the collagen was re-suspended in a strong acetic acid solution (33%). To decrease the acidity, the collagen mixture was enclosed in a dialysis membrane and submerged overnight in a dilute acetic acid solution (3%). Finally, the collagen solution's pH was neutralized (pH 7), and the solution was cast into a tray for air-drying at room temperature. This process resulted in the formation of a thin collagen film over four days [7].

Antibiotic standard preparation

Doxycycline hydrochloride (DOX), obtained from Himedia Laboratories Pvt. Ltd., India, was used for the preparation of the antibiotic stock solution. A quantity of 100 mg of DOX was dissolved in 1 ml of sterile distilled water by vortexing was used for subsequent experimental use [7].

Preparation and characterization of Delphinium denudatum silver nanoparticles (DdAgNPs)

Dd roots were sterilized by washing with double-distilled water and then dried in the shade at room temperature before being ground into a fine powder. To prepare the extract, 4 g of this powder was mixed with 40 ml of sterile double-distilled water and heated for 2 h at 60°C. The extract was then filtered using Whatman No. 1 filter paper. AgNPs were synthesized by combining 1.5 ml of the plant extract with a 30 ml solution of 1 mM silver nitrate. The reduction of pure silver ions (Ag+) to form AgNPs was monitored by measuring the solution's UV-visible spectrum (300 nm to 700 nm) using a Cecil spectrophotometer. High-resolution transmission electron microscopy (HRTEM) (JEOL JEM 2100, 200 kV resolution) was used to examine the size and shape of the resulting DdAgNPs. ImageJ software was then employed to calculate the average diameter of the nanoparticles [14].

In vivo studies

All animal experiments were performed using the protocol recommended by the Institutional Animal Ethics Committee (IAEC) of Sathyabama Institute of Science and Technology, Chennai (IAEC approval number: SU/CLATRI/IAEC/XI/106/2018). The effects of DdAgNPs, BTC, DOX, and combinations of these treatments on wound healing were investigated in Wistar albino rats. Male albino Wistar rats were divided into four groups and given different treatment conditions, Group-1 (control without any treatment for injury) Group-2 (treatment with 200μg DdAgNPs) Group-3 (treatment with a combination of 200μg DdAgNPs: 500μg BTC), and Group-4 (treatment with 200μg DdAgNPs: 500μg BTC: 15μg DOX solution). The rats were kept in groups for a week before the research and then housed individually with a 12-h light/dark cycle at 25 ± 1º C. They were given regular rodent feed from M/s Hindustan Lever Ltd., Mumbai, India, and water ad libitum [15].

Surgical procedure and dressings

 After injection of general anesthesia (ketamine-50 mg/kg body weight and xylazine-10 mg/kg body weight), the neck area below the back of the rat was shaved aseptically. A 1 × 1 cm full-thickness incision was made and each group was treated according to their treatment conditions. Every four days, the dressing material was changed. At 4-, 8-, and 12-day post-injury development, one rat from each group was euthanized to collect granulation tissue, which was refrigerated until analysis. Periodic observation of the wound contraction area, histopathology, and immunohistological examination were performed to assess rat wound healing [16].

Measurement of the rate of contraction and re-epithelialization by the Planimetric method

A digital snapshot is taken at a 10 cm distance at four-day intervals to provide visual evidence of the wound-healing process. The time taken for the wound samples to be fully re-epithelialized was recorded. The contraction ratio and surface area were determined using the conventional planimetric approach, by locating the lesion on a clear drawing sheet. The following mathematical equation was employed to determine the wound's closure rate [17].

where n = number of days (4th, 8th, 12th, and 16th day).

Histological and immunohistochemical analysis

To assess regeneration, skin samples were collected from the remodeled tissue at the wound site and the adjacent healthy dermis, which was within 2 mm of the wound margin, after euthanasia. These samples were embedded in paraffin, dehydrated using graded ethanol, and fixed in formalin. Subsequently, sections of 4 µm thickness were cut from the paraffin-embedded samples. To evaluate the extent of epidermal and dermal regeneration, tissue sections were subjected to hematoxylin and eosin (H&E) staining for histological examination. For immunohistochemical analysis, paraffin-fixed tissue sections were rehydrated using xylene followed by a series of ethanol solutions. These sections then underwent blocking with 5% bovine serum albumin in Tris-buffered saline (TBS, pH 7.4) for 2 h. After the blocking step, tissue sections were incubated with rabbit polyclonal IgG targeting rat COX, diluted 1:500 in TBS, at 4°C overnight. After the overnight incubation, the sections were washed three times with TBS to remove excess antibodies. This comprehensive protocol enables the assessment of granulation tissue regeneration through both histological examination and immunostaining for specific COX markers [18].

## Results

The result of this study has provided significant insights into the wound-healing properties of DdAgNPs, BTC, and DOX and their combined treatment in Wistar albino rats. Promising effects regarding wound closure, histological changes, and modulation of inflammatory response were observed.

Extraction of collagen from bovine Achilles tendons

The total weight of the dry collagen obtained was 10.263±0.82 g, indicating a yield of 0.82% for the bovine collagen sample.

Preparation and characterization of DdAgNPs

**Figure 1:**
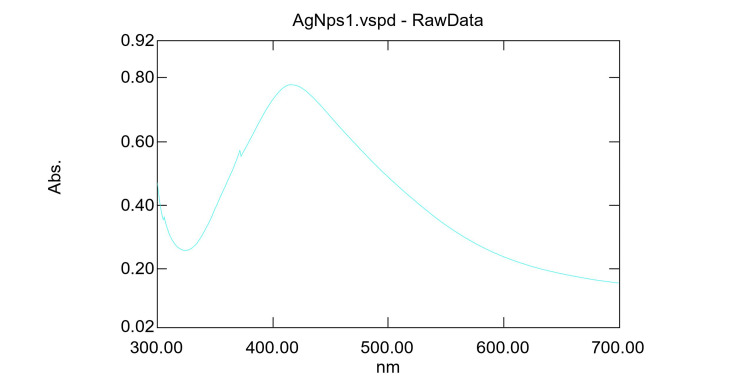
The formation of silver nanoparticles was confirmed using UV-visible spectroscopy, and an absorption peak was observed at 416 nm, indicating their presence and formation.

**Figure 2:**
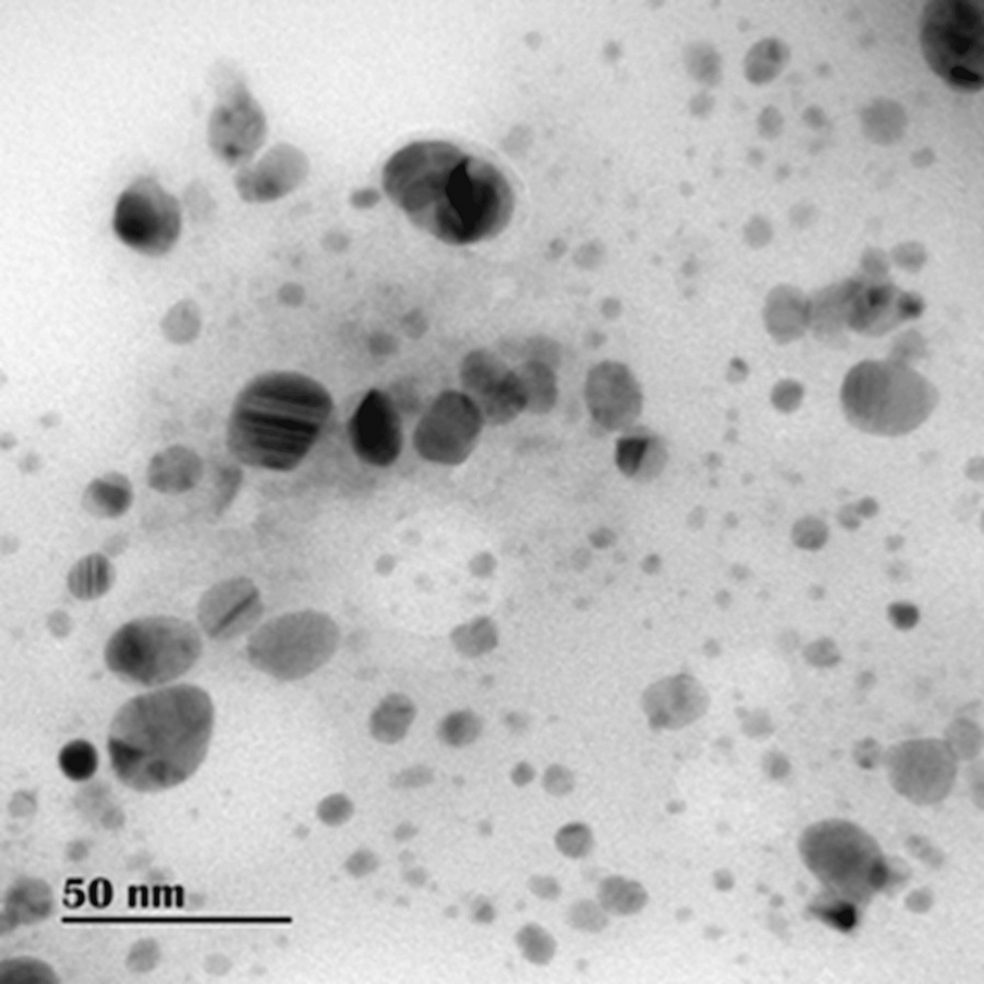
HRTEM images of DdAgNPs: Analysis by HRTEM revealed that the silver nanoparticles were polydisperse and spherical, with sizes up to 35nm ± 10.42, highlighting their heterogeneous distribution and stable spherical morphology. HRTEM: High-resolution transmission electron microscopy; DdAgNPs: Delphinium denudatum silver nanoparticles

The addition of aqueous extract to AgNO_3_ solution gradually turned yellowish brown. In contrast, no color change was observed in the root extract without AgNO_3_. In UV-visible spectroscopy, the absorption spectrum of silver colloids exhibited a peak at 416 nm, indicating the formation of AgNPs (Figure 1). The DdAgNPs analyzed using HRTEM were found to be polydisperse and spherical with particle sizes up to 35nm ± 10.42 (Figure 2).

In vivo wound-healing studies

**Figure 3:**
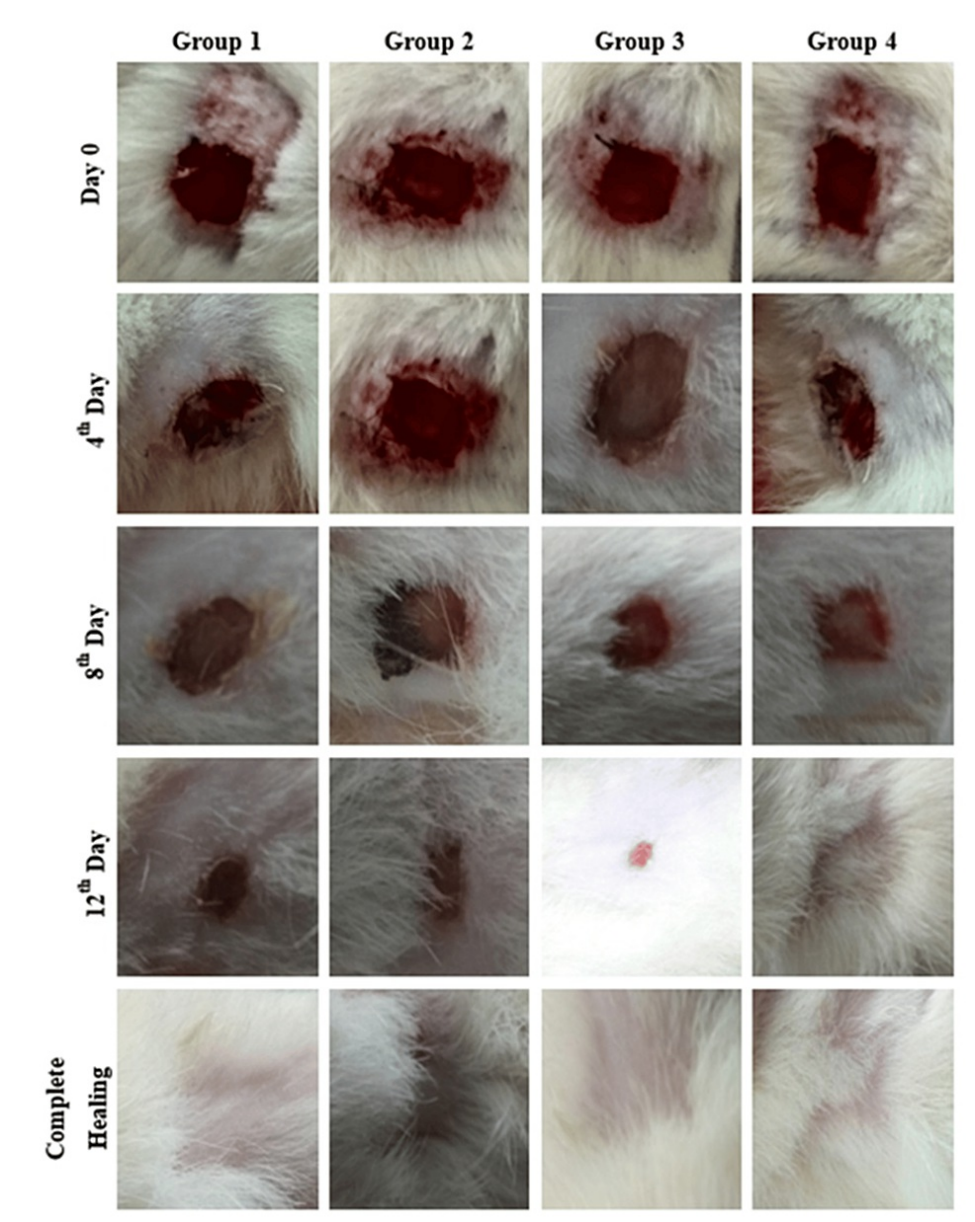
Photographic images showing the healing pattern of excisional wounds taken: Photographs taken at a distance of 10 cm documented wound healing, and planimetric studies were conducted every four days. Group 4, treated with DdAgNPs:BTC:DOX, showed faster healing than the other groups. Complete closure was achieved on day 10 with this treatment, outperforming others and the control group. DdAgNPs: Delphinium denudatum silver nanoparticles; BTC: bovine tendon collagen; DOX: doxycycline

Photographs were taken at a distance of 10 cm to provide evidence of in vivo wound healing scenes in all animals at four-day intervals. The results revealed that experimental Group 4 treated with DdAgNPs: BTC: DOX healed faster than the other groups (Figure 3), and the results showed that the groups were significantly different on a given day. The control group showed a slower rate of wound shrinkage at various time intervals. By day 14, both DdAgNPs and DdAgNPs:BTC groups exhibited similar levels of wound contraction leading to complete closure by day 16, while the DdAgNPs:BTC:DOX treated group showed significant improvement in wound contraction by day 9. The group showed a steady increase in wound contraction, reaching complete closure by day 10 three days earlier than the DdAgNPs:BTC group and eight days earlier than the control group. DdAgNPs:BTC and DdAgNPs groups required 14 and 15 days for complete recovery, respectively, while the control groups required nearly 16 days.

Histological observation

**Figure 4:**
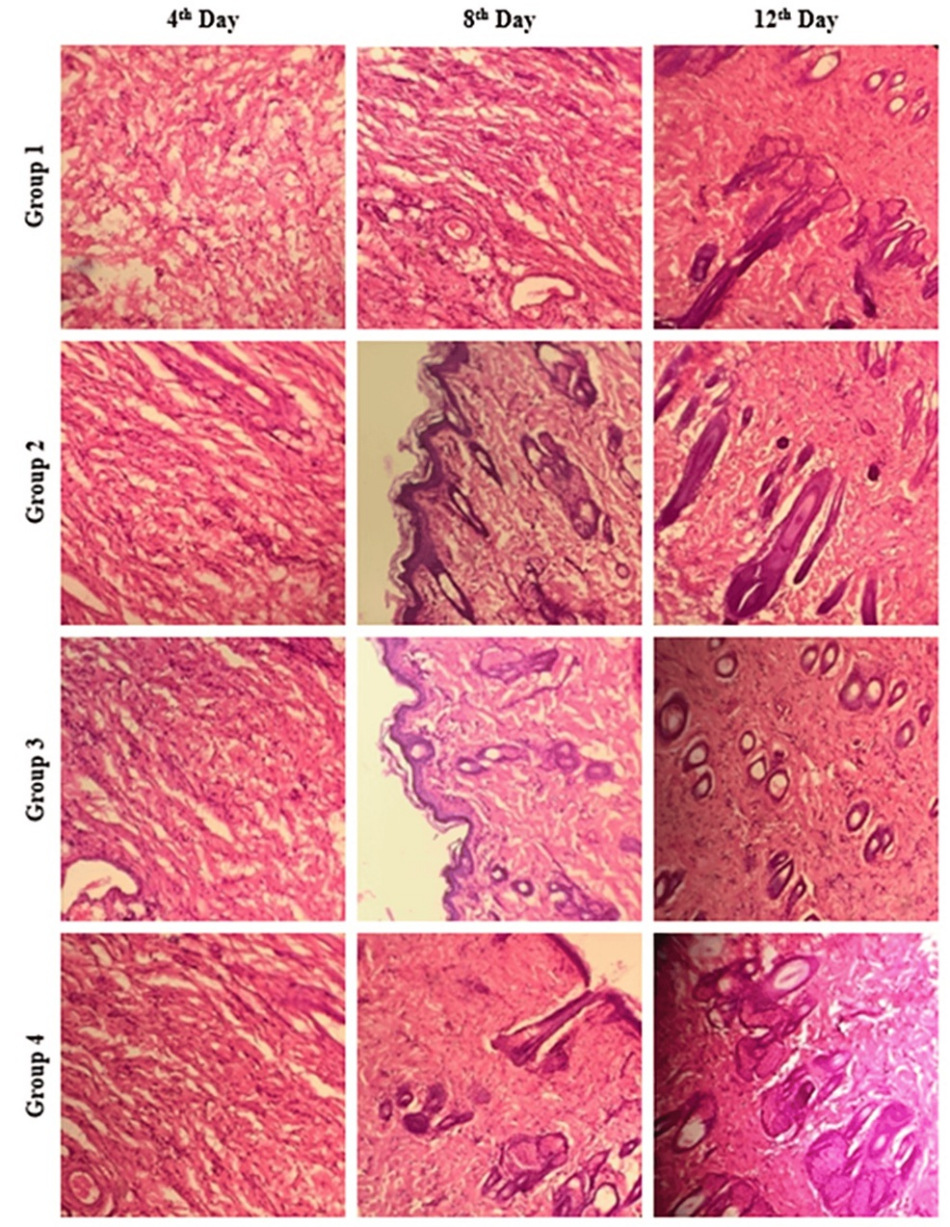
Histological examination of wound tissues after H&E staining: Histological examination using H&E staining demonstrated complete healing on day 12 in the DdAgNPs:BTC:DOX-treated group compared with others. DdAgNPs: Delphinium denudatum silver nanoparticles; BTC: bovine tendon collagen; DOX: doxycycline

Histological analysis using H&E staining was performed on tissue samples from all control and experimental groups at days 4, 8, and 12 post-wounding. This analysis aimed to assess key features of wound healing, including epithelial and connective tissue formation, inflammatory response, fibroblast proliferation, and collagen deposition (Figure 4). At day 4, both control and treated groups exhibited the presence of epithelium and connective tissue in the H&E-stained sections. By day 8, moderate inflammatory infiltration was observed in the DdAgNPs, DdAgNPs:BTC, and control wounds compared to the DdAgNPs:BTC:DOX group, which displayed epithelial and connective tissue with minimal inflammation. Additionally, the presence of blood vessels, connective tissue, and lymphocytes in the DdAgNPs:BTC:DOX group suggested that the DOX-linked scaffold facilitated faster healing by suppressing a prolonged inflammatory phase. Furthermore, improved wound margins were observed in the DdAgNPs:BTC:DOX group compared to the others. These findings collectively indicate a superior and accelerated healing process mediated by the DdAgNPs:BTC:DOX treatment. Notably, by day 12, complete wound closure was achieved only in the DdAgNPs:BTC:DOX group, demonstrating significantly better outcomes compared to the DdAgNPs, DdAgNPs:BTC, and control groups.

Immunohistochemical analyses of COX-2

**Figure 5:**
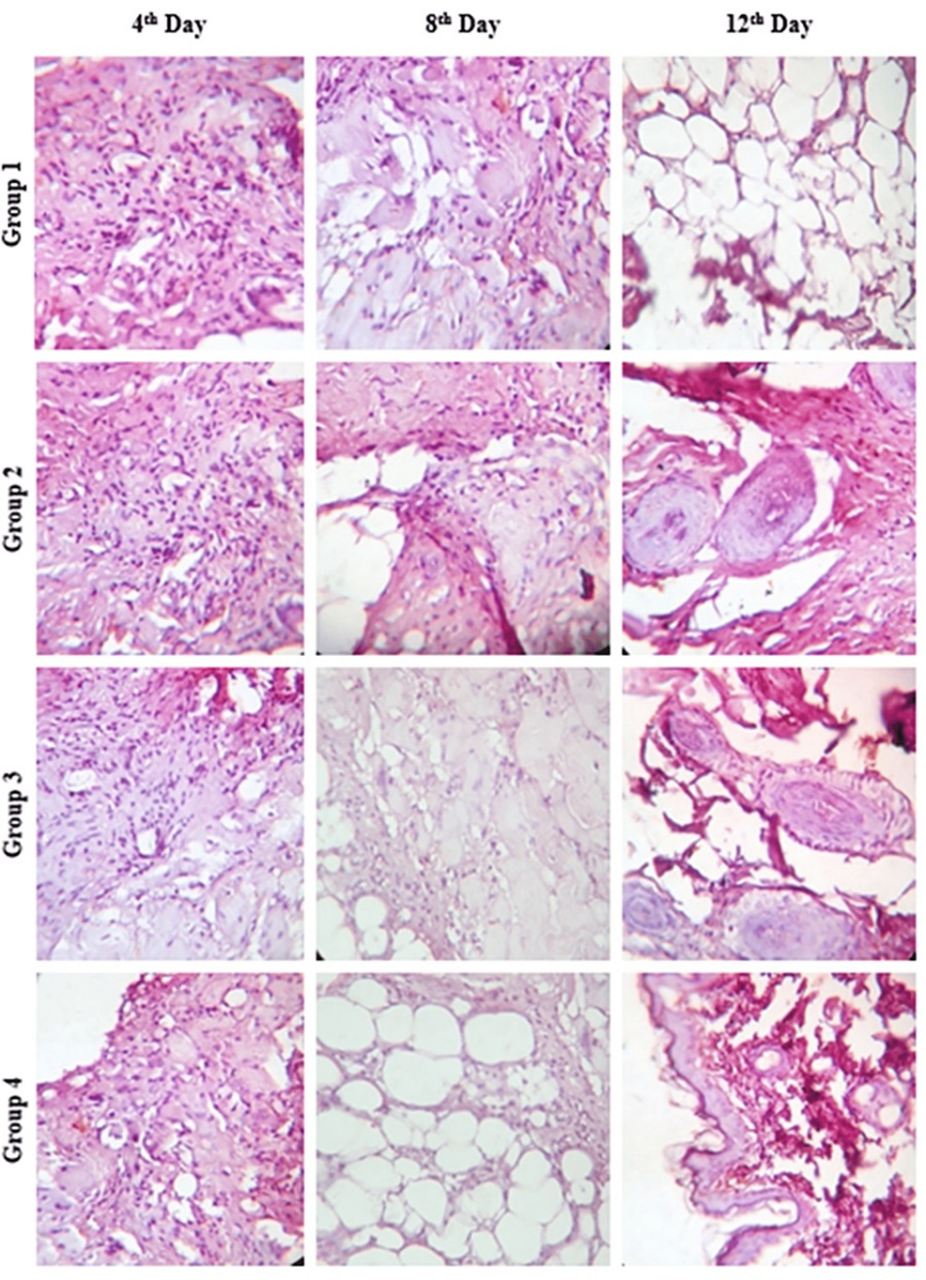
COX-2 immunohistochemical staining of sections of wound: COX-2 expression dynamics in wound healing: impact of treatment groups including DdAgNPs, DdAgNPs:BTC, and DdAgNPs:BTC:DOX. DdAgNPs: Delphinium denudatum silver nanoparticles; BTC: bovine tendon collagen; DOX: doxycycline

Initially, the control group showed higher levels of COX-2 expression, indicating inflammation in the early stages of wound healing. However, by day 8, COX-2 expression decreased in all treatment groups, suggesting resolution of the inflammatory phase. Notably, treatments including DdAgNPs and DdAgNPs:BTC led to a reduction in COX-2 expression, although not as significantly as the control group. A significant decrease in COX-2 expression was observed in the group treated with DdAgNPs:BTC:DOX, indicating a synergistic effect of this combination treatment. These results emphasize the potential of targeting COX-2 expression to promote wound healing. In addition, DdAgNPs:BTC:DOX showed faster efficacy in modulating inflammation and aiding wound healing (Figure 5).

## Discussion

Collagen from animal tendons primarily type I collagen acts as a promising agent with biological properties including higher cell adhesion capacity is less antigenic, and has been utilized in biomedical applications such as wound dressing, tissue engineering constructs, and cosmetics [19]. Three methods for extracting collagen include neutral salt-solubilized, pepsin-solubilized, and acid-solubilized [20]. The acid-solubilized method is a potent non-enzyme alternative to native collagen, with high amino acid content [21]. In this study, acetic acid was used to extract acid-soluble collagen from the bovine tendon, previously, collagen extraction from a variety of different sources, primarily sheep tail tendons, was attempted and a mass of 10.263 g of collagen was obtained from this investigation, offering important quantitative insight into the experimental result. According to the study of Paladini et al., Alpinia calgarata silver nanoparticles showed a sharp peak at 420nm by UV-Vis spectroscopy analysis and TEM results showed spherical nanoparticles with an average size of 27 nm [22]. Biosynthesized DdAgNP characterization carried out using UV-Vis showed a peak at 416 nm, HRTEM images showed that AgNPs are mostly spherical in shape and the particle size range is 35± 10.42 nm.

In our previous paper, we reported that XRD analysis of Dd AgNPs confirmed the crystalline nature of silver nanoparticles. FTIR analysis revealed that polyols and phenols were primarily responsible for this reduction. Upon combining the past and present physico-chemical analysis of Dd AgNPs, it was found that these nanoparticles are small, crystalline, spherical, and possess bioactive capping agents that could be useful in biomedical applications [8].

For an ideal wound dressing material, it should have antibacterial activity to inhibit the pathogens in the wound area. AgNPs with smaller diameters have been shown to have a stronger antimicrobial impact than those with greater diameters, and they also exhibit higher antibacterial activity than their bulk counterparts [23]. We previously reported on the antibacterial activity of DdAgNPs in a study [7], which served as a basis for further investigation into their potential in wound healing research. The wound healing ability of guar gum/curcumin-stabilized silver nanoparticle hydrogels showed complete healing of wounds only on day 16 [24]. Similarly, research on the use of sponge-like chitosan-loaded silver nanoparticles bio-catalyzed by idaurin in wound healing was also attempted, which resulted in complete wound healing only after day 16 [25]. This study found that inflammatory cells were decreasing in the 4th, 8th, and 12th-day sections of wound healing, indicating greater healing. The DdAgNPs:BTC:DOX-treated group significantly accelerated wound healing, promoting collagen deposition and faster regeneration. This treatment did not contribute to scar formation in the wounded areas, making it an effective alternative for scarless deep wound healing. However, application of DdAgNPs:BTC:DOX resulted in complete wound healing on day 12, 4 days earlier than the previous study. The molecular mechanism of COX-2 therapy was used to improve fracture repair. Our study found that COX-2 expression decreased in the wound tissue after various treatment conditions, with the control group showing higher expression on day 4. Wound healing may be impaired by epidermal COX-2 depletion, and COX-2 inhibition reduces scar formation. The results of the study corroborate earlier reports that COX-2 inhibition can reduce scar deposition following cutaneous damage. The study presents a novel hybrid dressing material as DdAgNPs:BTC:DOX, for faster wound recovery. The highlight of the study includes a combination of AgNPs, bovine tendon collagen, and doxycycline enhances wound healing by providing antimicrobial action, supporting structural integrity, and reducing inflammation. Silver nanoparticles offer broad-spectrum antimicrobial effects, bovine tendon collagen promotes extracellular matrix formation and tissue regeneration, and doxycycline reduces inflammation by inhibiting COX-2 expression, accelerating the healing process. Furthermore, the bias in this study is exclusive use of Wistar albino rats, which may not represent other models or humans, and dosing regimens optimized for rats that may not translate to human subjects. Controlled laboratory conditions also differ from real-world environments, potentially affecting outcomes. Limitations include species-specific differences that hinder generalizability to humans, a focus on short-term effects without assessing long-term impacts, and a lack of genetic and demographic diversity in the rat model. These factors underscore the need for further research to validate and extend these findings to human clinical applications. The challenges of these findings in clinical practice involve scaling up the production of the combination therapy, navigating regulatory approvals, and conducting extensive human trials to ensure safety and efficacy. Addressing these steps is crucial for the successful application of this treatment in clinical settings

## Conclusions

This study investigates the use of collagen, DOX, and biosynthetic AgNPs using Dd to cure wounds in albino Wistar rats. In vivo wound healing was observed, with the experimental group treated with DdAgNPs: BTC: DOX exhibiting faster healing compared to other groups. The group also showed faster collagen deposition and epithelial regeneration, accelerating wound healing. The DdAgNPs:BTC:DOX scaffold significantly up-regulated wound healing processes, reducing COX-2 expression. The results suggest that these nanoparticles could potentially accelerate wound healing in rats. Overall, the findings of this study indicated that DdAgNPs:BTC:DOX has improved wound healing properties with advantages like quicker wound healing, i.e., in 12 days, and inhibition of COX-2 production. More research is needed to determine the functions of growth factors like COX-2 throughout the wound healing process.
